# Community Dynamics in the Mouse Gut Microbiota: A Possible Role for IRF9-Regulated Genes in Community Homeostasis

**DOI:** 10.1371/journal.pone.0010335

**Published:** 2010-04-23

**Authors:** Claire L. Thompson, Markus J. Hofer, Iain L. Campbell, Andrew J. Holmes

**Affiliations:** 1 School of Molecular and Microbial Biosciences, University of Sydney, Sydney, Australia; 2 Department of Neuropathology, University of Marburg, Marburg, Germany; Columbia University, United States of America

## Abstract

**Background:**

Gut microbial communities of mammals are thought to show stable differences between individuals. This means that the properties imparted by the gut microbiota become a unique and constant characteristic of the host. Manipulation of the microbiota has been proposed as a useful tool in health care, but a greater understanding of mechanisms which lead to community stability is required. Here we have examined the impact of host immunoregulatory phenotype on community dynamics.

**Methods and Findings:**

Denaturing gradient gel electrophoresis was used to analyse the faecal bacterial community of BALB/c and C57BL/6 mice and C57BL/6 mice deficient for either type I interferon (IFN) signalling (IRF9 KO mice) or type I and type II IFN signalling (STAT1 KO mice). Temporal variation was found in all mouse strains. A measure of the ability for a community structure characteristic of the host to be maintained over time, the individuality index, varied between mouse strains and available data from pigs and human models. IRF9 KO mice had significantly higher temporal variation, and lower individuality, than other mouse strains. Examination of the intestinal mucosa of the IRF9 KO mice revealed an increased presence of T-cells and neutrophils in the absence of inflammation.

**Significance:**

The high temporal variation observed in the gut microbiota of inbred laboratory mice has implications for their use as experimental models for the human gut microbiota. The distinct IRF9 and STAT1 phenotypes suggest a role for IRF9 in immune regulation within the gut mucosa and that further study of interferon responsive genes is necessary to understand host-gut microbe relationships.

## Introduction

The gastro-intestinal tract of mammals is colonised with a diverse range of micro-organisms. In recent years evidence has accumulated to support the idea that the community structure of the gut microbiota is a major contributor to the phenotype of the host animal. This evidence comprises three distinct streams: Firstly, available gut community dynamics studies have shown high temporal constancy within, and distinct composition between, adult individuals [1]–[3]. Secondly, the activity of gut microbes directly contributes to a variety of physiological and metabolic processes that are important to host function [4]–[6]. There is experimental evidence that the integration of microbial activity into host metabolism, in conjunction with maintenance of individual community differences, gives rise to microbiota-linked phenotypes [7]–[9] including obesity [10] and drug response [11], [12]. Thirdly, gnotobiotic animal studies show that molecules of microbial origin are essential for host developmental pathways [13] and that different microbial strains can engender distinct host responses [14]. Collectively these observations suggest important roles for the gut community in health, however defining a healthy gut community is difficult.

Microbial communities do not show absolute constancy of structure and furthermore different members are predicted to turn over at different rates. Consequently, observed differences in composition between two gut community samples at any one time point will reflect both transient differences (such as those due to high turnover populations, changes in abundance or allochthonous populations) and sustained differences (due to autochthonous populations). It is the sustained differences that are most relevant to microbiota-linked phenotypes, such as obesity. The phenomenon of sustained host-specific differences in microbial communities (referred to from here on as individuality) reflects the property of ecological resilience and has two distinct aspects, temporal constancy and constraints on composition. Understanding individuality is important since it also represents a barrier to manipulating gut microbial communities. Resilient communities resist change and if change results from disturbance they tend to return to the previous state. We postulate that different aspects of the host immune system may contribute to the different aspects of individuality and community stability. To explore this, we examined mice that differ in their capacity to regulate immune responses measuring both the constancy of community structure in individual mouse gut communities and the differences in community structure between mice, to derive a measure of the relative importance of sustained and transient differences in gut community between animals that we refer to as the individuality index.

Adult mice of two different genetic backgrounds (BALB/c & C57BL/6) were analysed. These strains have normal functional immune systems but have well defined differences in their immunophenotype including different MHC haplotypes and immune responses that are biased to either a Th1 like (C57BL/6) or a Th2 like (BALB/c) immune response [15]. We also examined two strains of C57BL/6 mice deficient in interferon (IFN) signalling pathways. Studies in the caecal epithelia of gnotobiotic mice have shown that interferon-responsive genes are among the host genes that respond more strongly to a bacterial community than to mono-specific colonisations [14] and the interferon pathway is a major part of the immune response to many bacterial, parasitic and viral infections [16]–[20].

STAT1 KO mice [16] lack the signal transducer and activator of transcription 1 (STAT1) which is essential for the signalling of type I (IFN-α/β) and type II IFNs (IFN-γ) [21]. In contrast, interferon regulatory factor 9 (IRF9) is primarily involved in type I IFN signalling and not IFN-γ signalling [22]. Consequently, IRF9 KO mice [17] are predicted to be impaired in type I IFN but not in IFN-γ signalling. Constancy was assessed for each individual mouse at both daily and 5 day time scales. The individuality index was shown to vary between strains of different immunophenotype supporting the hypothesis that immune function is an important part of gut community individuality. Surprisingly the strongest effect was observed in IRF9 KO mice, suggesting previously unrecognised regulatory pathways may be involved.

## Materials and Methods

### Animals and sample collection

All mice were housed under specific pathogen free conditions within the same room in the animal facilities at the University of Sydney and handled according to the guidelines and approved protocols of the University of Sydney Animal Ethics Committee. Mice were given food and water *ad libitum*. A total of 19 individual mice were used for this study from two different genetic backgrounds and two gene deficient mouse strains. These were wild type (WT) BALB/c (n = 5) and C57BL/6 mice (n = 5) and STAT1 KO mice (n = 5) [16] and IRF9 KO (n = 4) [17], both on a C57BL/6 background. All mice of the same strain were co-housed in a filter top cage with the exception of the IRF9 KO mice which were split into two cages (total of 5 cages for the four strains). All mice were sampled at 17 weeks of age in the main study. In a preliminary study, C57BL/6 mice were also sampled at 10 weeks of age. Each individual mouse was specifically tagged and hence each faecal sample could be assigned to a particular mouse. Faecal samples were collected every five days for 20 days with additional samples collected daily between days 15 to 20 (Refer to supplementary Table S1). A total of 216 faecal samples were obtained from 19 individuals. Samples were collected directly from the animal upon defecation and immediately frozen at −20°C, prior to DNA extraction.

### DNA extraction

Extraction of DNA from faecal samples was carried out using the FastPrep system (Bio101, La Jolla, CA, USA) with modifications as described previously [23], [24]. Briefly, faecal pellets were homogenised in 500 µl TE buffer (10 mM Tris, 1 mM EDTA, pH 7.5) prior to extraction. Cells were lysed with one 5mm glass bead and 0.6 g of 150–600 µm glass beads (Sigma Aldrich, St Louis, MO, USA) as per the protocol for the FastDNA Spin Kit for soil (Bio101). The yield of DNA obtained from the mouse faecal samples was between 10 ng to 100 ng per µl as determined by agarose gel electrophoresis.

### PCR

PCR primers F-968-GC and R-1401 [25] were used to amplify the V6–V8 region of the 16S rRNA gene. Each 25 µl reaction volume contained 1× Thermopol buffer (New England BioLabs, USA), 5 mM deoxynucleoside triphosphates (New England BioLabs), 20 pmoles F-968-GC, 10 pmoles R-1401, 1U Taq Polymerase DNA (New England BioLabs) and 1 µl of faecal DNA. The program used was as follows: 1 minute of initial denaturation at 94°C, followed by 30 cycles of denaturation (94°C for 30 seconds), annealing (56°C for 30 seconds) and extension (72°C for 1 minute) with a final extension for 7 minutes at 72°C.

### DGGE

DGGE analysis was performed using the DCode system (Bio-Rad Laboratories, USA). Electrophoresis was done using on a 16 cm×16 cm 1 mm thick gel that contained 8% polyacrylamide (ratio of acrylamide to bisacrylamide was 37.5∶1) in 1× TAE buffer (40 mM Tris-acetate 1 mM EDTA; pH 7.4). A gradient of 40–70% denaturant was used to separate PCR fragments where 100% denaturant was defined as 7M urea and 40% (v/v) formamide. The gels were run at 80V for 16 hours at 60°C and silver stained as described in Sambrook and Russell [26]. Gels were scanned using a GS-800 calibrated densitometer (Bio-Rad Laboratories, USA). The digitised gel images were analysed using Quantity One (version 4.6.1; Bio-Rad). The software was used to detect bands by normalising against total intensity data for each lane. Bands with a minimum density of 5% were detected in each lane and bands were matched using a match tolerance of 2%. A similarity matrix was constructed using Dice's similarity coefficient. This is defined as 
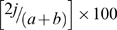
 where *j* is the number of bands in common between two lanes and (*a*+*b*) is the total band number of both lanes. Reproducibility was assessed by electrophoresis of independent amplifications of the same DNA sample. This was found to be very high for within-gel analyses [24], but not between gels as previously reported in other studies [27], [28]. Thus, all pairwise comparisons of DGGE fingerprints were between samples that were run on the same gel.

### Statistical Analysis

Analysis of statistical significance was done using Prism (version 3.0; GraphPad Software, San Diego, CA). A two-tailed Student's t test was used to compare averages of the Dice coefficient when two groups of mice were compared. Where more than two groups were compared, the Kruskal-Wallis nonparametric ANOVA test was used to assess significance.

In order to describe the impact of inter-individual differences relative to temporal variation a simple calculation that we term the individuality index (*II_t_*) was derived.
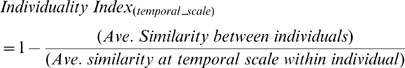
A positive value signifies that the level of individual-to-individual variation is greater than temporal changes within individuals at that scale of observation. A negative value denotes that change over time is greater than inter-individual variations. The individuality index was calculated for all mouse strains using data obtained at 5 day and daily sampling scales.

### Tissue processing for histology

Mice were euthanized and the large intestines were removed for histological and immunohistochemical examination. Tissues were fixed in PBS-buffered 4% paraformaldehyde (pH 7.4, Sigma-Aldrich) for 48 hours at 4°C prior to being embedded in paraffin. For histology and immunohistochemistry, 5 µm thick sections were prepared.

### Histology and immunohistochemistry

Haematoxylin and eosin (H&E) stained sections were examined to assess gross anatomical features of the large intestines of mice from the four different strains. Stained H&E and unstained sections were provided by the Histopathology Laboratory, Department of Pathology, University of Sydney. Primary polyclonal antibodies specific for T-cells (human anti-CD3, Dako, Botany, Australia), activated macrophages (mouse anti-Iba1, Wako Chemicals, USA) and neutrophils (mouse anti-Gr-1, Caltech, USA) were used for immunohistochemistry at a dilution of 1∶200. Paraffin sections were deparaffinized in xylene and rehydrated in a series of graded ethanol. For staining against CD3, slides were pretreated with proteinase K (Sigma Aldrich, 10 µg/ml, 15 min at 37°C). For staining against Gr-1 and Iba1, slides were immersed in 10mM sodium citrate buffer (pH 6.0) at a sub-boiling temperature for 10 minutes, followed by cooling at room temperature for 30 minutes. Following blocking with 10% normal goat serum in PBS for 30 minutes slides were incubated overnight at 4°C with the primary antibodies. Slides were washed in PBS-T (PBS+0.1% Tween20, pH 7.4) three times for 5 minutes and a biotinylated secondary antibody (Vector Labs, Burlingame, CA 1∶200, 45 minutes) and horseradish peroxidase coupled streptavidin (Vector Labs, 1∶200, 30 minutes) were added successively. Nova Red (Vector Labs, USA) was applied as the immunoperoxidase substrate according to the manufacturer's instructions. Sections were counterstained with haematoxylin (Sigma-Aldrich, USA), dehydrated, cleared and coverslips were mounted prior to examination by bright field microscopy (Leica DM4000B, Leica Germany). Digital images were taken using a Spot Flex camera and Spot V4.5 software (Diagnostic Instruments, USA).

For quantification of immuno-positive cells 10-high-power fields (40× objective) per section were counted and mean and standard error of the mean (s.e.m). determined. Statistical significant differences were determined by one-way-ANOVA and Bonferroni's multiple comparison test using Prism 4 (GraphPad Software, San Diego, USA).

## Results

### The gut microbiota of adult mice had relatively low constancy of composition

**Figure 1:**
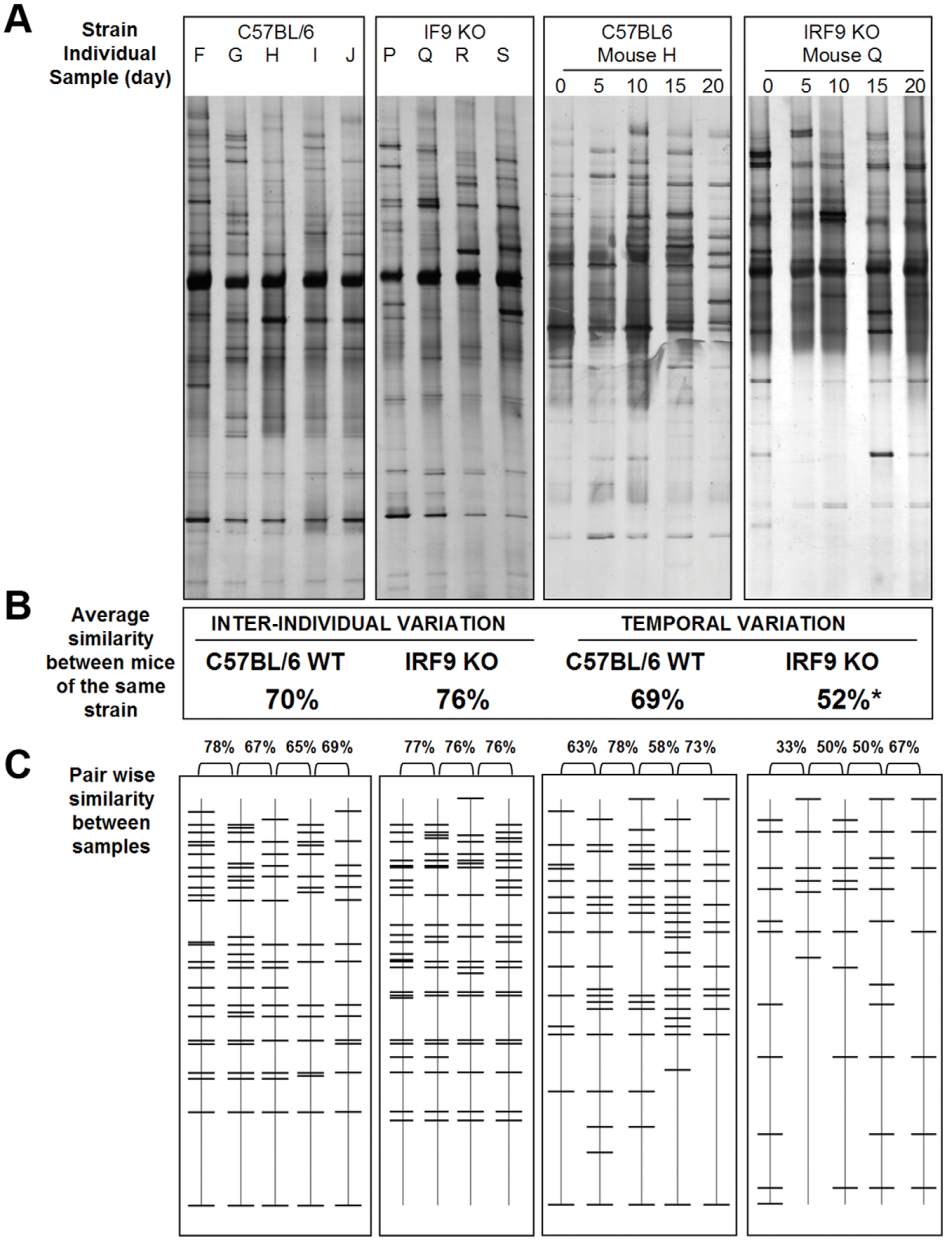
Inter-individual and within-individual temporal variation in faecal communities. (A) DGGE profiles of the overall faecal community of individual mice from the C57BL/6 and IRF9 KO mouse strains and examples of change over time when sampled at 5 day intervals for one mouse from each of the two strains. (B) Average similarity of DGGE profiles for the indicated comparisons of all mice of the same strain. (C) Schematic representation of bands detected in each profile after image analysis (see methods) and calculated pairwise similarity. * indicates significant difference at P<0.05 (see Fig 2).

We monitored temporal variation in the composition of the gut microbiota of healthy mice that were housed under controlled conditions. An estimate of community constancy was determined as the average similarity of DGGE profiles of faecal samples obtained at 5 day or at daily intervals for each individual. Figure 1 shows examples of DGGE profiles over time in an individual mouse from the C57BL/6 and IRF9 KO mouse strains. All mice showed changes in community structure over time when sampled at either 5 day or daily intervals. Mean temporal variation was less for samples taken on a daily basis than mean temporal variation for samples taken every 5 days (P<0.02), indicating the importance of the temporal scale of sampling in determining community constancy. For the C57BL/6 mice this experiment was performed twice, at ages 10 and 17 weeks and no significant difference was seen for either daily or 5-day sample scales (data not shown).

**Figure 2:**
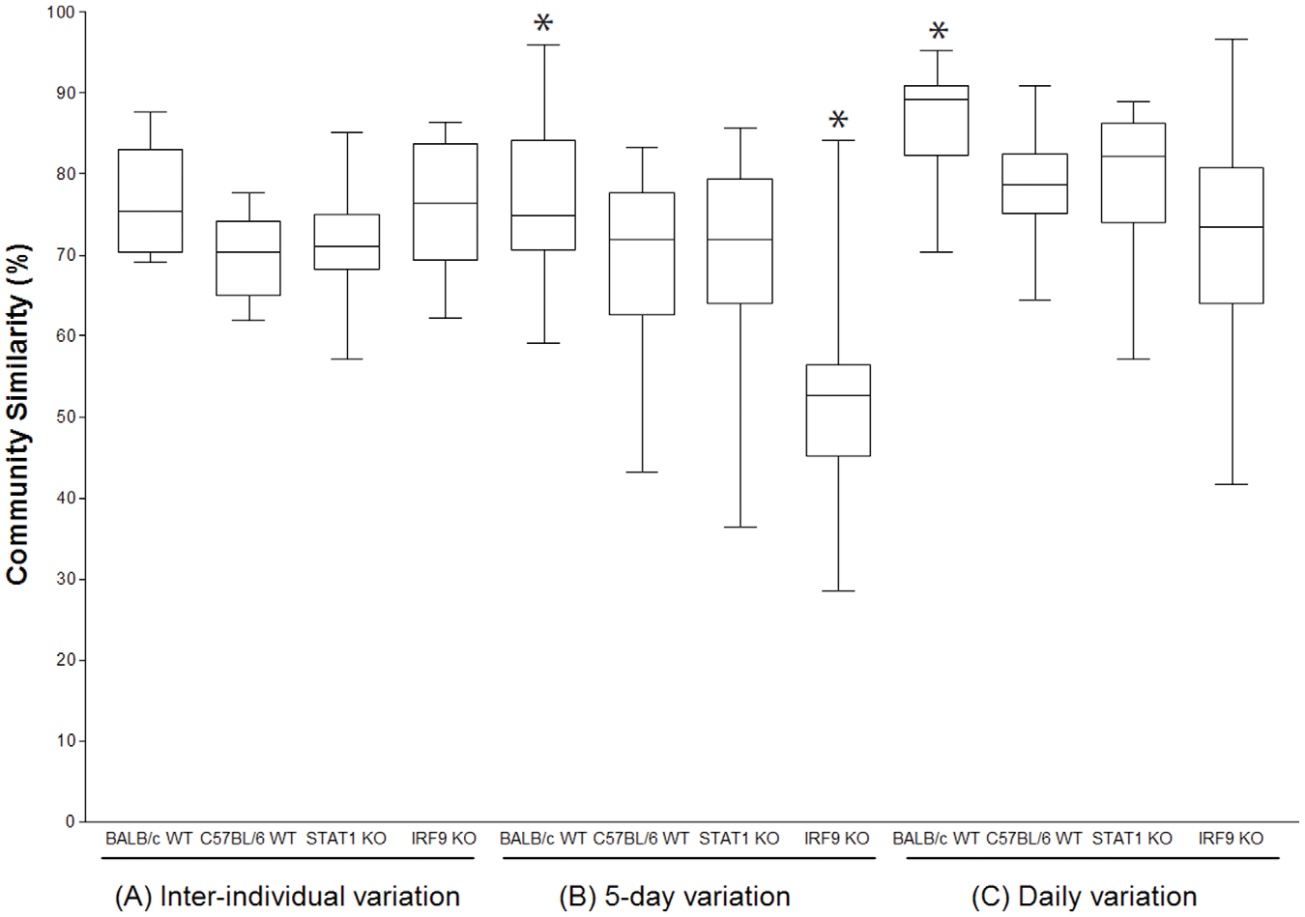
Box and whiskers graph of similarity coefficients calculated for the DGGE profiles of faecal communities of mice. The box extends from the 25th percentile to the 75th percentile, with a line at the median (the 50th percentile). The whiskers extend above and below the box to show the highest and lowest values. (A) Inter-individual variation was similar for each strain and is calculated by comparing the faecal DGGE profiles of each mouse of the same strain at a single time point (17 weeks of age). Temporal variation (bacterial community turnover at each day) was calculated comparing pair-wise similarity between adjacent faecal profiles at 5 day (B) and daily intervals (C). All mice showed change in community structure over time. The highest level of constancy was observed in the BALB/c mice. The lowest level of constancy was observed in the IRF9 KO mice. * indicates significant difference at P<0.05 for strains compared at the same temporal scale. Significance was determined using a Kruskal-Wallis nonparametric ANOVA test.

The degree of change over time varied between mouse strains (Figure 2). Comparisons between the WT mice indicated that temporal stability was significantly higher in the BALB/c mice compared to C57BL/6 mice when sampled at daily and 5 day intervals (P<0.02). Temporal variation in IRF9 KO mice was significantly higher compared to all other mouse strains when sampled at 5 day intervals (P<0.01). When sampled daily, the IRF9 KO mice also had the highest measure of temporal variation but this was not significantly different from its WT equivalent. In contrast, the degree of temporal variation in the other IFN-signalling deficient mouse strain, STAT1 KO, was not significantly different from the wild type mice of the same genetic background when sampled at either 5 day or daily intervals (P>0.05).

### Mice have low individuality of gut community structure

Individual-to-individual variation within each of the four mouse strains was compared by analysing the overall faecal community at a single time point (day 0; all mice aged 17 weeks). All DGGE fingerprints were non-identical indicating that each mouse had a distinct faecal community (Figure 1). The individuality index provides a framework to predict the relative contributions of transient and resident populations when comparing differences between two communities. *II_N_* values were calculated for pigs based on published data [24] and for all four mouse strains in this study (Table 1). A negative *II_N_* index for a system predicts that differences between communities predominantly reflect transient variation at that observational scale and a positive index predicts observed differences reflect intrinsic properties of the system. For our DGGE data set, the only time scale at which any mouse strains had positive *II_N_* was 1 day and at the longer time scale of 5 days all 4 mouse strains had negative *II_N_*. In contrast calculations of the pig *II* from previously published data are very strongly positive at time scales of either 1 or 5 days. Of particular interest was that the IRF9 mice had a more negative *II_N_* value at both time scales. This implicates IRF9 function in maintenance of the individuality of the gut microbiome in mice.

**Table 1:** Parameters of community variation for each mouse strain sampled at 5 day and daily intervals compared to the pig gut microbiota.

*Strain*	*Ave. Similarity between individuals*	*Ave. Similarity within individual over time*	*Individuality index*
II_(5-day)_ BALB/c WT	79.9	77.3*	−0.03
II_(5-day)_ C57BL/6 WT	69.8	68.8	−0.01
II_(5-day)_ STAT1 KO	71.4	69.7	−0.02
II_(5-day)_ IRF9 KO	76.4	56.6*	−0.35
II_(daily)_ BALB/c WT	76.9	86.2*	0.11
II_(daily)_ C57BL/6 WT	69.9	79.0	0.12
II_(daily)_ STAT1 KO	71.9	78.8	0.09
II_(daily)_ IRF9 KO	76.4	72.6	−0.05
II_(5-day)_ Pig^a^	51.6	90.6	0.43
II_(daily)_ Pig^a^	51.6	97.6	0.47

WT (wild type).

*significant difference to strains sampled at the same time interval (P<0.05).

a Data from the DGGE analysis of faecal samples obtained daily from a >30-day old pig (Thompson *et al.*, 2008).

### IRF9 KO mice show an increased presence of T-cells and neutrophils in the intestinal mucosa and lymphatic nodules

**Figure 3:**
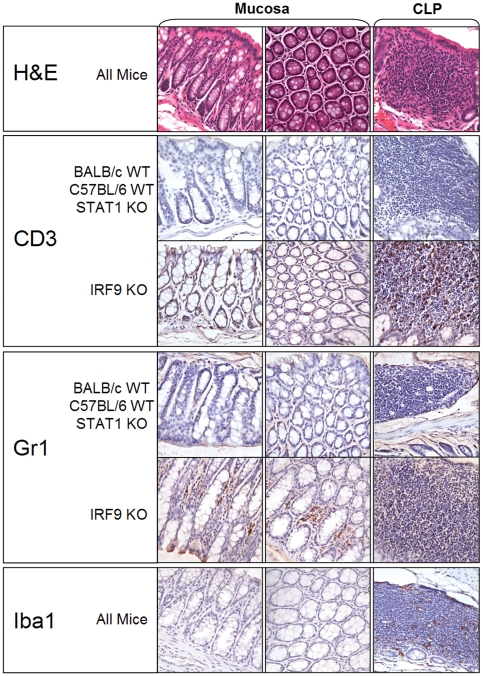
Representative images showing mucosal differences of IRF9 KO mice. The images shown are from C57BL/6 WT and IRF9 KO mice (Tissue sections from BALB/c and STAT1 KO mice were equivalent to C57BL/6 WT mice in all cases and are not shown). Relative to the other strains, IRF9 KO mice showed a marked increase in presence of brown stained CD3 positive cells (T-cells) and Gr-1 positive cells (neutrophils) in both the mucosa and lymphatic nodules of the large intestine. No discernible differences were seen between the four mouse stains in H&E- or Iba1-stained (macrophage) sections of the large intestine. Images taken at 63× magnification.

Haematoxylin and eosin (H&E)-stained tissue sections of the large intestine showed no difference in the overall structural features between mice (Figure 3). The mucosa was intact and goblet cells were similar in number and appearance. Organised lymphatic tissue in the mouse large intestine is arranged as intramucosal and submucosal follicles, termed colonic lymphoid patches (CLP). In all mice investigated, these were of similar size, number and appearance. We found no evidence for an inflammatory response such as accumulations of lymphocytes around a blood vessel (perivascular lymphocyte cuffs).

In order to further characterize the cells in the CLPs and to determine the number of intra-epithelial lymphocytes (IELs) immunohistochemistry for CD3 (T-cells), Gr-1 (neutrophils) and Iba1 (macrophages) was performed. No differences were observed between the WT mice of both strains and the STAT1 KO mice. However, IRF9 KO mice showed an increased presence of CD3 positive cells (T-cells) and Gr-1 positive cells (neutrophils) in the mucosa (IELs) and CLPs (Figure 3) as compared with the other mouse strains (Table 2). These histological features were present in all of the IRF9 mice and none of the mice showed signs of illness during the course of the study that could explain this variation. No difference was observed in the number or localisation of the Iba1 positive macrophages.

**Table 2:** Frequency of immuno-positive intraepithelial cells.

	C57Bl/6 (WT)	Balb/c (WT)	STAT1 KO	IRF9 KO
CD3+ cells	14.3±2.4^a^	29.3±2.8	25.6±6.5	118.8±12.9 ***^b^
Gr1+ cells	3.3±0.8	2.0±1.1	4.0±1.2	37.8±9.2 ***
Iba1+ cells	3.1±0.5	7.5±6.5	5.3±1.9	3.8±2.8

a Mean ± s.e.m.

b ***: p<0.001 compared to C57Bl/6, Balb/c and STAT1 KO mice.

## Discussion

Our understanding of biological variation in the gut ecosystem is central to exploring many aspects of the host-microbiota relationship, yet has received relatively little attention. Temporal variation is particularly relevant to understanding emergent properties of microbial activity in the host system. A constant community structure is likely to provide continuity of microbial metabolic activities for the host and give rise to stable microbe-dependent phenotypic traits. Identification of specific controls on the structure of the gut community is anticipated to ultimately give rise to mechanisms for re-engineering the composition of poorly performing communities. Host factors such as the immune system are postulated to have a role in promoting the stability of a gut community with host-specific composition.

### The gut microbiota of mice exhibits relatively low individuality

Maintaining a stable gut community ensures that the properties imparted by the gut microbiota become a unique and constant characteristic of a host. Furthermore, the extent to which these properties apply to the gastro-intestinal systems of different mammals remains to be determined. In studies of the human and pig gut microbiota, individuality is high and inter-individual differences are far greater than changes in community structure within one individual over time [1], [2], [29], [30]. In contrast, all mice in this study showed a low degree of individuality and temporal change was only less than the inter-individual variation when daily samples were analyzed (Table 1). This was true of all mice regardless of genotype or deficiency in IFN-signalling pathway components. The consequence of this is that microbiota-influenced traits in mice will potentially show stochastic variation over time. Indeed recently profiles of microbially-derived metabolites were reported to show considerable variability over time in healthy adult mice [31] and in another study populations of mice that were split into separate housing facilities showed changes in community fingerprints over time that correlated with their environment [32].

With the exception of IRF9 KO mice that were kept in two separate cages, mice of each strain were co-housed and we cannot exclude that variation may result from differences in microbial exposure from cage-mates. However, as the degree of variability between the IRF9 KO mice was independent of housing, this suggests that this factor may play a minor role. In addition, temporal variability was observed in all mouse strains regardless of genotype, housing or differences in family history. The relative instability of the mouse gut microbiota observed here may be a biological property of mice or could reflect environmental (specific pathogen free housing conditions) or biological (inbred populations) aspects of our experimental design. Instability may also result from anatomical differences. When compared to humans and pigs, the mouse colon is shorter and the caecum is larger, relative to colon size [33], [34]. This results in differences in the time taken by food to pass through the intestine and available data suggest average transit time is shorter for mice (10 hours) than humans (2–4 days) [33], [35]. It is conceivable that higher turnover of faecal material influences community dynamics. An alternate hypothesis is that instability is a product of the inbred nature or housing conditions of laboratory mice. Inbreeding is considered to reduce immune function and increase disease propensity and therefore may change the way the host responds to the gut microbiota [36]–[38]. The microbial load received by laboratory mice housed under pathogen free conditions is reduced as food and bedding are autoclaved. The major microbial exposure of individual mice is to the microbiota of other mice through contact such as coprophagy [39]. Since exposure to microbes under these conditions is confined largely to those that have also been seen by other mice of similar genotype there may be less ecological isolation. It is conceivable that the temporal variation observed here reflects dynamics within a larger meta-community and might not be observed in outbred populations with non-sterile food.

### The degree of temporal variation was dependent on genotype

Some differences in community dynamics were evident between strains. In this study, BALB/c mice had significantly higher constancy compared to C57BL/6 mice (Figure 2). These strains have well defined differences in the regulation of their immune responses including distinct MHC haplotypes and immune responses that are biased to either a Th1 like (C57BL/6) or a Th2 like (BALB/c) immune response [15]. This may potentially influence the way each mouse strain responds to the gut microbiota [40], [41]. The skewing of the immune response to either the Th1 or Th2 type response results in higher susceptibility to autoimmune or allergic disease, respectively [42]–[46]. These diseases are often accompanied by disturbances to the community dynamics of the gut microbiota and indicate a possible role for the immune system in regulation of the gut microbiota [47], [48]. The genotypic differences between C57BL/6 and BALB/c mice evidently underpin different host response to bacterial antigens [40], [41], [49], [50] and may explain the difference in stability between strains.

Interferons have a role in a variety of immune responses and disruption of IFN function renders mice highly susceptible to microbial infections. Studies in gnotobiotic mice have shown that the host epithelial tissue response to co-colonization by commensal bacteria is the synergistic induction of interferon-responsive genes [14]. Furthermore, recent studies have suggested the IFN-signalling molecules IRF9 and STAT1 to be critical mediators of B cell responses including antibody isotype switching and the expression and activation of nucleic acid sensing Toll-like receptors (TLRs) [51]. These are functions which have been linked to the regulation of the gut microbiota [52], [53]. Here we tested mice of two distinct interferon pathway deficient genotypes for their ability to regulate community dynamics. STAT1 is essential for signalling in both type I and II IFN pathways whereas, IRF9 is involved in type I IFN signalling where it acts in interaction with STAT1 and STAT2 in a heterotrimeric complex termed ISGF3 [54], [55]. An ISGF3-independent role for IRF9 has been proposed, but remains unclear [22]. Therefore it was surprising to find the IRF9 KO mice had the highest temporal variability when compared to the other mouse strains and no observable phenotype in the STAT1 KO mice (Figures 1 & 2). This phenomenon was consistent with the distinctive mucosal histology of IRF9 KO mice whereby increased numbers of T-cells and neutrophils dispersed in the intestinal mucosa in the absence of obvious inflammation were evident (Figure 3). Since temporal variation and loss of individuality was increased only in the case of IRF9 deficiency, it is likely that IRF9-regulated gene expression is involved in host-microbe crosstalk. It is worth noting that in a recent study aimed at identifying host genes specifically targeted by commensal (as opposed to pathogenic) bacteria, IRF9 was one of the most strongly up-regulated host genes by the oral commensal bacterium *Streptococcus salivarius* K12 [56].

### Conclusions and implications of relative instability in the mouse gut microbiota

Gut microbes contribute to physiological, metabolic and developmental outcomes of the host and hence are an integral part of the host phenotype. The high temporal variation and lack of predictable stable differences observed in the gut microbiota of inbred laboratory mice has implications for their use as experimental models particularly in the study of phenotypes influenced by microbial activity. Our observations indicate that laboratory mice do show a degree of individuality in their gut microbiota, but this is significantly less than that of humans. The higher turnover of the mouse gut community relative to humans (or pigs), suggests that links between microbial community structure and host phenotype will be even stronger in humans than they are in mice. It also means that individual mice may exhibit microbiota-related phenotypic variation during the course of longer studies with implications for study design. This area requires further investigation and is particularly important in the case of mouse models that have been developed to look at health issues closely related to the human gut system including inflammatory bowel disease models in IL-2 and IL-10 KO mice [57], [58] that develop colitis in the presence of a gut microbiota [59], [60] and inbred mouse models used for studying drug metabolism and obesity [9], [11].

The concept of the community individuality index provides a basis to recognize a ‘tipping point’ when community stability becomes too low to contribute meaningfully to host phenotype. We found laboratory mice did show gut microbiota individuality, hence they are valid models for emergent phenotypes. However, mouse gut community individuality was low relative to other systems and we predict use of inbred laboratory mice as models for gut microbiota-related characteristics of the host will underestimate the importance of such effects in humans. The low stability of the mouse gut microbiota observed here could be a consequence of the inbred nature of laboratory mice or an inherent feature of mouse biology. Our observation that individuality was lowest in the IRF9 KO mice suggests IRF9 KO mice will be a useful model to assess the importance of stability for microbial-influenced phenotypes and that identification of IRF9-regulated genes may result in targets for manipulation of the gut microbiota composition.

## Supporting Information

Table S1 Faecal sample collection from four mouse strains.
(0.03 MB PDF)
