# Supplementary material for: Community Dynamics in the Mouse Gut Microbiota: A Possible Role for IRF9-Regulated Genes in Community Homeostasis
Source: PLoS One. 2010 Apr 23;5(4):e10335. doi: 10.1371/journal.pone.0010335 (PMC2859068; doi:10.1371/journal.pone.0010335)
Supplement: Table S1 — Faecal sample collection from four mouse strains. (0.03 MB PDF) [file pone.0010335.s001.pdf]

Table S1 Faecal sample collection from four mouse strains

Background	***BALB/c***					***C57BL/6***													
Strain	**Wild Type**					**Wild Type[1]**					**STAT1 KO**					**IRF9 KO**			
Individual	**A**	**B**	**C**	**D**	**E**	**F**	**G**	**H**	**I**	**J**	**K**	**L**	**M**	**N**	**O**	**P**	**Q**	**R**	**S**
Litter/Cage	**1**	**1**	**1**	**1**	**1**	**2**	**2**	**2**	**2**	**2**	**3**	**3**	**3**	**3**	**3**	**4**	**4**	**5**	**5**
Day 0																			
1																			
2																			
3																			
4																			
5																			
6																			
7																			
8																			
9																			
10																			
11																			
12																			
13																			
14																			
15																			
16																			
17																			
18																			
19																			
20																			

[1]The same individuals were sampled twice; at 10 weeks and 17 weeks of age. Samples were collected every 5 days initially and then daily from day 15. Samples collected at day 20 were used for both 5 day and daily sample sets.
